# Emphasis should be placed on identifying and reporting research priorities to increase research value: An empirical analysis

**DOI:** 10.1371/journal.pone.0300841

**Published:** 2024-03-22

**Authors:** Yicheng Gao, Zhihan Liu, Rui Cao, Yingdi Liao, Yuting Feng, Chengyuan Su, Xinmiao Guan, Rui Fang, Yingjie Deng, Wenyuan Xiang, Junchang Liu, Yuanyuan Li, Yutong Fei

**Affiliations:** 1 Centre for Evidence-Based Chinese Medicine, Beijing University of Chinese Medicine, Beijing, China; 2 Institute of Excellence in Evidence-Based Chinese Medicine, Beijing University of Chinese Medicine, Beijing, China; 3 Beijing GRADE Centre, Beijing, China; 4 Kunming Traditional Chinese Medicine Hospital, Kunming, China; 5 Affiliated Hospital of Traditional Chinese Medicine, Xinjiang Medical University, Wulumuqi, China; Universidad del Cauca, COLOMBIA

## Abstract

**Objectives:**

To compared the presentation of research priorities in the GRADE (Grading of Recommendations Assessment, Development, and Evaluation) clinical practice guidelines (CPGs) developed under the guidance of the GRADE working group or its two co-chair, and the Chinese CPGs.

**Methods:**

This was a methodological empirical analysis. We searched PubMed, Embase, and four Chinese databases (Wanfang, VIP Database for Chinese Technical Periodicals, China National Knowledge Infrastructure and Chinese Biomedical Literature Database) and retrieved nine Chinese guideline databases or Society websites as well as GRADE Pro websites. We included all eligible GRADE CPGs and a random sample of double number of Chinese CPGs, published 2018 to 2022. The reviewers independently screened and extracted the data, and we summarized and analyzed the reporting on the research priorities in the CPGs.

**Results:**

Of the 135 eligible CPGs (45 GRADE CPGs and 90 Chinese CPGs), 668, 138 research priorities were identified respectively. More than 70% of the research priorities in GRADE CPGs and Chinese CPGs had population and intervention (PI) structure. 99 (14.8%) of GRADE CPG research priorities had PIC structures, compared with only 4(2.9%) in Chinese. And 28.4% (190) GRADE CPG research priorities reflected comparisons between PICO elements, approximately double those in Chinese. The types of research priorities among GRADE CPGs and Chinese CPGs were mostly focused on the efficacy of interventions, and the type of comparative effectiveness in the GRADE research priorities was double those in Chinese.

**Conclusions:**

There was still considerable room for improvement in the developing and reporting of research priorities in Chinese CPGs. Key PICO elements were inadequately presented, with more attention on intervention efficacy and insufficient consideration given to values, preferences, health equity, and feasibility. Identifying and reporting of research priorities deserves greater effort in the future.

## Introduction

In the development of clinical practice guidelines (CPGs), the formation of recommendations requires a comprehensive consideration of the quality of evidence, desirable and undesirable anticipated effects, health equity and other dimensions. The quality of the evidence was heavily considered as a key determinant dimension [1, 2]. However, in practice, evidence retrieval, particularly of high-quality evidence, often proves challenging [3–5]. Despite these limitations, recommendations were typically formulated based on the best available evidence. For the research gap, guideline developers tend to report research priorities through summarization and prioritization.

Simultaneously, another contrary situation arises where high-quality evidence has already existed and is robust enough that would unlikely be overturned in the future. In such cases, research saturations, a special kind of priority, should be presented to discourage further research, thus averting research waste. Furthermore, some guideline manuals also include research priorities as a form of research recommendations [6–10]. To be more precise, research priorities are not solely evidence gaps or knowledge gaps, but rather proposals for prioritized future research based on the current evidence.

The development of guidelines requires huge intelligence and financial input. Therefore, methodological rigor is crucial, and the guideline development group is also in the most suitable position to determine the research priorities.

Since the 1990s, China has developed a considerable number of CPGs [11], and the number has been increasing rapidly over time [12]. Serving as statement documents to guide Chinese clinicians in decision-making [13–15], CPGs has been playing a crucial and distinct role. The Grading of Recommendations Assessment, Development, and Evaluation (GRADE) approaches, developed by the GRADE working group that is led by world-leading evidence-based medicine experts, published in 2016, were considered a gold standard in guideline development, often provides a paradigm of high-quality methodology. Their structured and transparent approaches, including a fixed research priority dimension embedded in the framwork, offered valuable guidance for guideline development groups [16–18].

Although there were some studies on research gaps [19–23], the exploration of the research priorities in the guidelines was still inadequate, especially the systematic analysis of the form and content of the research priorities [24]. This study systematically investigated and compared the CPGs that were developed under guidance of the GRADE working group and the Chinese CPGs, to offer reference and guidance for the development and reporting of research priorities in the future.

## Methods

### Identification of research priorities

We defined the research priorities as the focus of the most important future research topics addressing the needs of developing guidelines or recommendations. We usually find them in the EtD (evidence to decision) framework, discussion, evidence summary, etc., and identify their common terms including: research priorities, future research, evidence gaps, further research, etc.

### Guidelines sources and searches

We searched two English databases (PubMed, Embase) and four Chinese databases (Chinese Biomedical Literature Database, China National Knowledge Infrastructure, Wanfang, and VIP Database for Chinese Technical Periodicals). We also searched the website (https://www.GRADEpro.org/) and methodological papers published by two co-chairs of the GRADE working group to find more GRADE CPGs. And we searched nine guideline databases or official websites of authoritative Chinese societies to find more Chinese guidelines (S1 File for the detailed search strategy).

### Eligibility criteria

The GRADE CPGs and the Chinese CPGs that were published from the 1st January 2018 to the 31st December 2022 were included. GRADE CPGs are defined as CPGs developed under the guidance of the GRADE working group or its two co-chairs. Chinese CPGs are CPGs published in Chinese. Number of included Chinese CPGs were designed to be a random sample with twice as much as the included GRADE CPGs. Older versions and duplicate published CPGs were ineligible.

### Data extraction and analysis

Two reviewers worked independently in pairs, the methods of systematic reviews of screening titles, abstracts, full text, and data extraction were strictly implemented. Unresolved differences were settled through consultation until consensus was achieved or by a third reviewer. We extracted the following information based on the standardized data extraction form designed in advance: (1) the basic characteristics of the guidelines, such as the type, scope and publication year, whether reported the research priorities, etc.; (2) the relevant information of the research recommendations, including reason, type (e.g., PICO, population, intervention, comparison, and outcome), structure, dimensions related to recommendations of research priorities, etc. Descriptive statistical analysis was used.

### Registration

We have registered at INPLASY with the registration number INPLASY202350083.

## Results

### Search results

**Fig 1:**
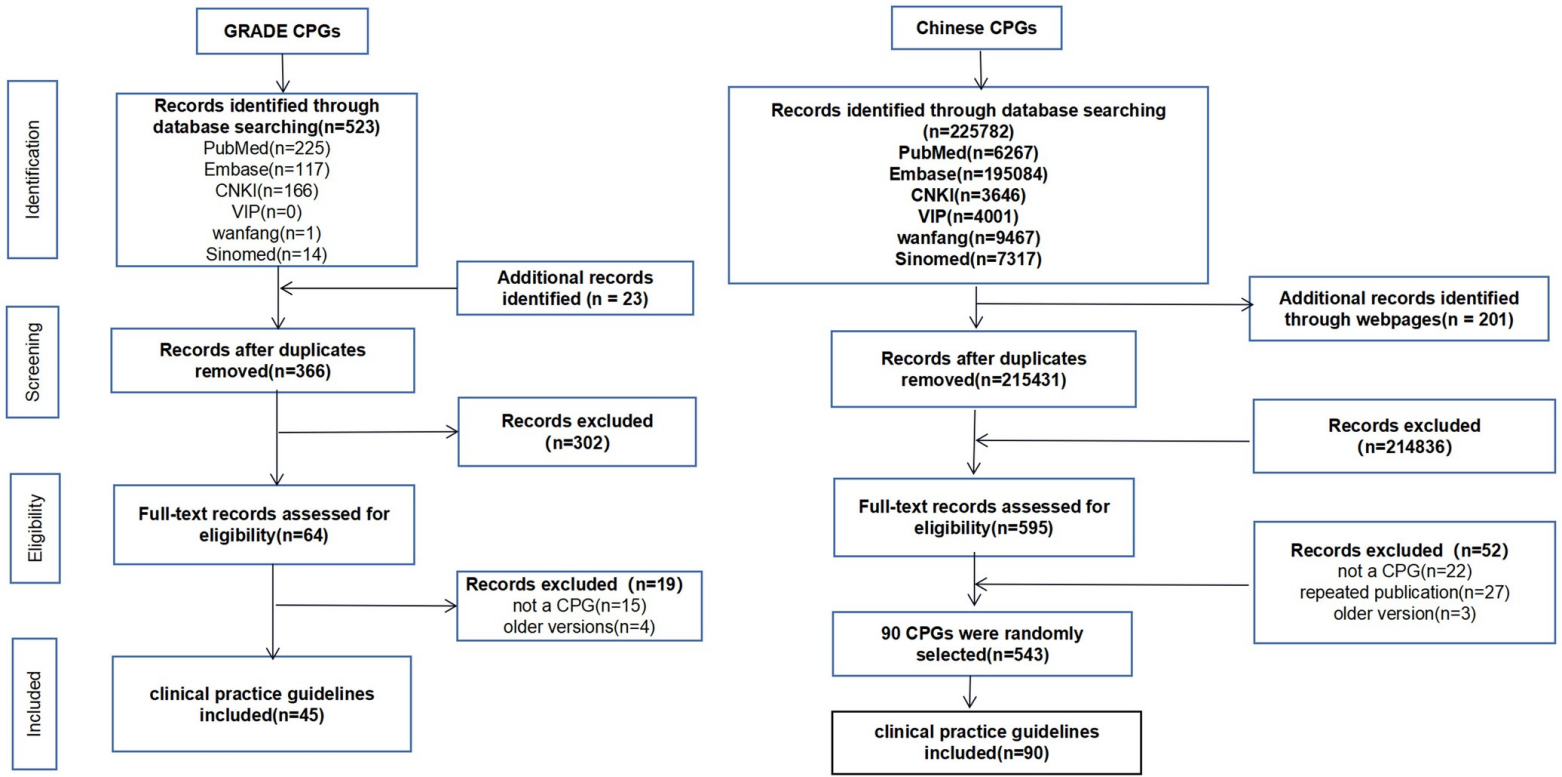
Clinical practice guidelines processing flow diagram.

The retrieval process for the GRADE CPGs and the Chinese CPGs were performed separately. For the GRADE CPGs, 523 records were retrieved from the databases, and an additional 23 records were obtained from other sources, resulting in 64 remaining records after removing duplicates and ineligible entries. Among these, four older versions and 15 records not meeting guideline criteria were excluded, leaving 45 CPGs included. Regarding Chinese CPGs, 225782 and 201 records were retrieved from databases and other sources respectively, 595 remained after removing duplicate and unqualified records. 3 older versions, 27 duplicate publications, 22 not guidelines were excluded. Finally, 543 Chinese CPGs were identified, of which 90 were randomly selected for inclusion (S2 File and Fig 1).

### Guideline characteristics

A total of 135 CPGs were included (45 GRADE CPGs and 90 Chinese CPGs). Among the GRADE CPGs, about 90% (40, 88.9%) reported research priorities, with more than half (22, 55%) orienting to the whole guidelines. In Chinese CPGs, only less than 30% (26, 28.9%) of the guidelines reported research priorities, of which more than 70% (20, 76.9%) were for the whole guidelines. Furthermore, ten (25.0%) of the GRADE CPGs reported more than 20 research priorities, while the Chinese CPGs all presented fewer than 20 (Table 1).

**Table 1:** Characteristics of included clinical practice guidelines N %.

Characteristics	GRADE CPGs (n = 45)	Chinese CPGs (n = 90)	Total (n = 135)
**Type of CPGs**			
Evidence-based CPGs	45(100.0)	83(92.2)	128(94.8)
Non-evidence-based CPGs	0(0.0)	7(7.8)	7(5.2)
Total	45(100.0)	90(100.0)	135(100.0)
**Scope of CPGs**			
Treatment or Prevention or Management	39(86.9)	87(96.7)	126(93.3)
Diagnosis or Screening	4(8.9)	2(2.2)	6(4.4)
Others	2(4.4)	1(1.1)	3(2.2)
Total	45(100.0)	90(100.0)	135(100.0)
**Publication year**			
2022	8(17.8)	21(23.3)	29(21.5)
2021	10(22.2)	21(23.3)	31(30.0)
2020	8(17.8)	16(17.8)	24(17.8)
2019	4(8.9)	20(22.2)	24(17.8)
2018	15(33.3)	12(13.3)	27(20.0)
Total	45(100.0)	90(100.0)	135(100.0)
**Report research priorities**			
Yes	40(88.9)	26(28.9)	66(48.9)
No	5(11.1)	64(71.1)	69(51.1)
Total	45(100.0)	90(100.0)	135(100.0)
**What the research priorities were oriented to**			
Guidelines as a whole	22(55.0)	20(76.9)	42(63.6)
Specific recommendation	13(32.5)	4(15.4)	17(25.8)
Guideline and recommendation	5(12.5)	2(7.7)	7(10.6)
Total	40(100.0)	26(100.0)	66(100.0)
**Location of the research priorities** ^a^			
Discussion of the guidelines	27(67.5)	22(84.6)	49(74.2)
Summary of the evidence under each recommendation	1(2.5)	5(19.2)	6(9.1)
Independent dimension under each recommendation	16(40.0)	0(0.0)	16(24.2)
Research recommendation^b^	1(2.5)	3(11.5)	4(6.1)
Total	40(100.0)	26(100.0)	66(100.0)
**Number of research priorities**			
≤5	17(42.5)	19(73.1)	36(54.5)
6–10	10(25.0)	4(15.4)	14(21.2)
11–20	3(7.5)	3(11.5)	6(9.1)
21–30	1(2.5)	0(0.0)	1(1.4)
≥31	9(22.5)	0(0.0)	9(13.6)
Total	40(100.0)	26(100.0)	66(100.0)

^a^ Some guidelines present research priorities at multiple places, so the total number of items is not 45, 90, 135.

^b^ Research recommendations as independent recommendation.

CPG, clinical practice guidelines; GRADE, Grading of Recommendations Assessment, Development and Evaluation; Chinese, Traditional Chinese Medicine.

### Structure and presentation form of research priorities in CPGs

The GRADE CPGs reported a total of 668 research priorities, while the Chinese CPGs reported only 138. As for the reasons for developing the research priorities, 515 (77.1%) GRADE CPG research priorities reported the lack of evidence, while the Chinese CPG research priorities further reported the lack of high-quality evidence (71, 51.4%). Analyzing the structure of research priorities, we found that over 70% of both GRADE CPGs and Chinese CPGs had structures (P, I), comprising 475 (71.1%) and 101 (73.2%) respectively. In contrast, there were 99 (14.8%) GRADE research priorities with PIC structures compared to only 4 (2.9%) in the Chinese CPGs. Notably, 190 (28.4%) GRADE CPG research priorities involved the comparison between PICO elements, approximately double those in Chinese CPG research priorities (Table 2).

**Table 2:** Structure and presentation form of research priorities N %.

Variable	GRADE CPG research priorities (n = 668)	Chinese CPG research priorities (n = 138)	Total (n = 806)
**Presentation forms**			
Clinical questions	91(13.6)	17(12.3)	108(13.4)
Narrative sentences	577(86.4)	121(87.7)	698(86.6)
**The reasons for presenting the research priorities**			
Lack of evidence, implicit	0(0.0)	38(27.5)	38(4.7)
Lack of evidence, explicit	515(77.1)	8(5.8)	523(64.9)
Lack of high-quality evidence, implicit	82(12.3)	10(7.2)	92(11.4)
Lack of high-quality evidence, explicit	62(9.3)	61(44.2)	123(15.3)
Further research is not recommended	1(0.1)	1(0.7)	2(0.2)
Not reported	8(1.2)	20(14.5)	28(3.5)
**Reflections of the PICO elements**			
P	11(1.6)	13(9.4)	24(3.0)
I	21(3.1)	4(2.9)	25(3.1)
PI	475(71.1)	101(73.2)	576(71.5)
PIO	10(1.5)	1(0.7)	11(1.4)
PIC	99(14.8)	4(2.9)	103(12.8)
PICO	0(0.0)	0(0.0)	0(0.0)
Not reflected	52(7.8)	15(10.9)	67(8.3)
**Whether the comparison between PICO PICO elements (such as I:C)**			
Yes	190(28.4)	20(14.5)	210(26.1)
No	478(71.6)	118(85.5)	596(73.9)

CPG, clinical practice guidelines; GRADE, Grading of Recommendations Assessment, Development and Evaluation; Chinese, Traditional Chinese Medicine; PICO, population, intervention, comparison, outcome.

We identified 1076 and 128 descriptions related to the research priorities in the GRADE CPGs and Chinese CPGs, respectively. The descriptions of the research priorities in the GRADE CPGs were concentrated, with approximately 60% (635, 59.0%) described as “research priorities/needs/agenda/are needed/question”, with "research priorities" alone accounting for 51.4% (553). In contrast, descriptions of Chinese research priorities were more scattered and relatively simple. The maximum number of “questions to be solved/studied/addressed” described was 43 (33.6%), followed by “further research / studies / clarified” and “future research / research should prioritize to address”, 28 (21.9%) and 20 (15.6%), respectively (S1 and S2 Figs).

### Focus of research priorities

Both the research priorities of the GRADE CPGs and the Chinese CPGs were clustered around efficacy of interventions, accounting for 42.1% (281) and 52.2% (72), respectively. Regarding the proportion of research priorities for comparative effectiveness, that is the condition if different interventions would need to be compared to address the research priority, GRADE research priorities were approximately double those in Chinese (accounted for 30.2% versus 15.2%). More than half of the research priorities in the GRADE CPGs and the Chinese CPGs reflected the dimension of desirable or undesirable anticipated effects, which are in the GRADE EtD framework for formulation of recommendations. The GRADE CPG research priorities considered a higher proportion of health equity and acceptability, while the Chinese is more concerned about the feasibility and quality of evidence (Table 3).

**Table 3:** Focus of research priorities in GRADE CPGs and Chinese CPGs N %.

Focus	GRADE research priorities (n = 668)	Chinese research priorities (n = 138)	Total (n = 806)
**Focus relevant to clinical features**			
Efficacy of interventions	281(42.1)	72(52.2)	353(43.8)
Comparative effectiveness ^a^	202(30.2)	21(15.2)	223(27.7)
Clinical assessment or clinical management	71(10.6)	17(12.3)	88(10.9)
Disease characteristics	30(4.5)	1(0.7)	31(3.8)
Intervention implementation details	25(3.7)	6(4.3)	31(3.8)
Subgroup of patients	23(3.4)	1(0.7)	24(3.0)
unspecified ^b^	32(4.8)	16(11.6)	48(6.0)
Others ^c^	4(0.6)	4(2.9)	8(1.0)
**Focus relevant to GRADE EtD dimensions for formulating recommendations** ^**d**^			
Desirable anticipated effects	101(15.1)	13(9.4)	114(14.1)
Only the general “effects” were mentioned	320(47.9)	76(55.1)	396(49.1)
Undesirable anticipated effects	98(14.7)	12(8.7)	110(13.6)
Values and preferences	16(2.4)	4(2.9)	20(2.5)
Health economics considerations	31(4.6)	10(7.2)	41(5.1)
Feasibility	4(0.6)	2(1.4)	6(0.7)
Acceptability	5(0.7)	0(0.0)	5(0.6)
Health equity	2(0.2)	0(0.0)	2(0.2)
Quality of evidence	0(0.0)	4(2.9)	4(0.5)

^a^ We classified a research priority as comparative effectiveness if different interventions or population would need to be compared to address the research priority.

More treatment or studies are needed

^b^ More treatment or studies are needed

^c^ Basic science research, intervention-standardized definition, decision aids, policy-making, specification.

^d^ Sometimes a research priority may focus on multiple dimensions of recommendations, so the total number of items does not equal 668,138,806.

CPG, clinical practice guidelines; GRADE, Grading of Recommendations Assessment, Development and Evaluation; Chinese, Traditional Chinese Medicine.

### Recommendations for the study methods and details of intervention implementation in the research priorities

**Fig 2:**
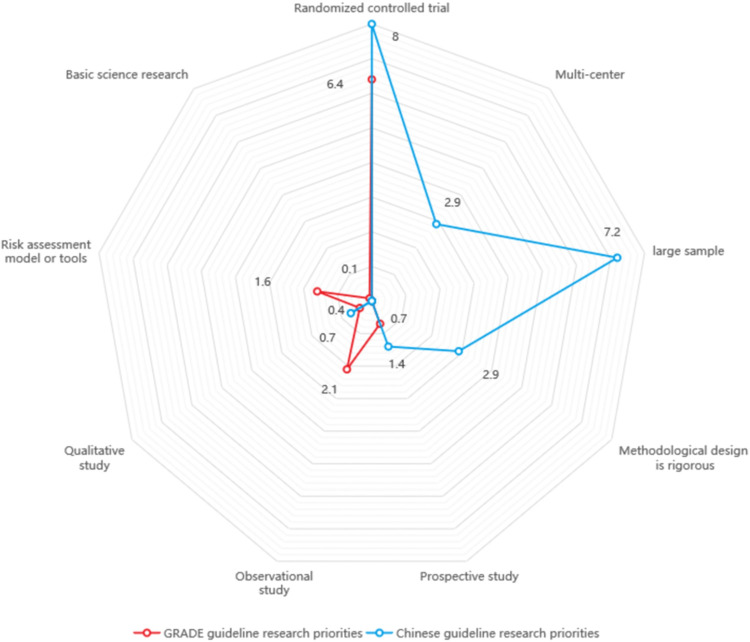
The percentage of research priority recommendations for the study methods.

**Fig 3:**
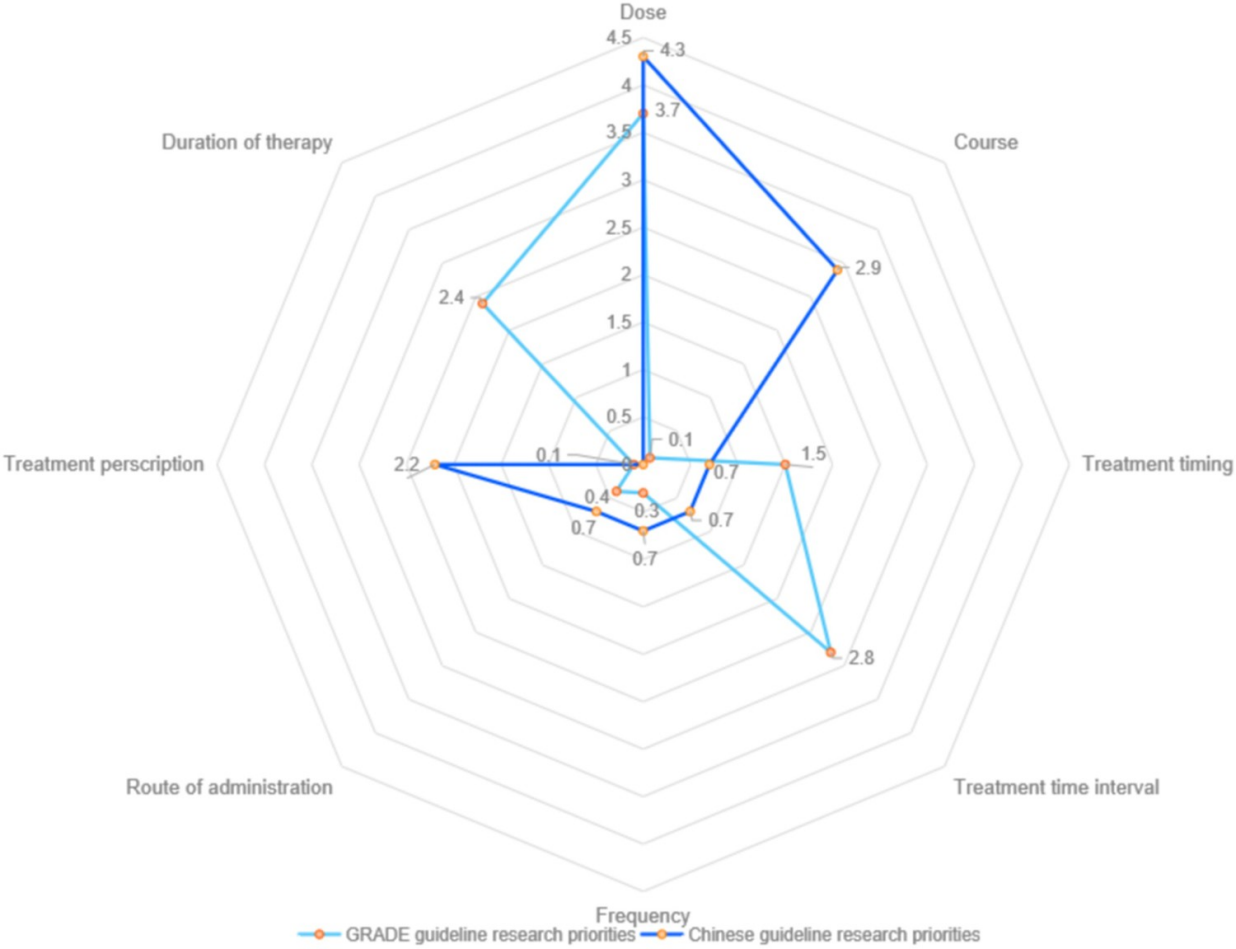
The percentage of details of intervention implementation.

The number of research priorities concerning recommended research methods among GRADE CPGs and Chinese CPGs was relatively small, with randomized controlled trials being the primary research method, accounting for 6.4% (43) and 8.0% (11), respectively. GRADE CPGs also recommends observational studies (14. 2.1%) and risk assessment model or tools (11, 1.6%), while Chinese CPGs mentioned multi-center (4, 2.9%), large sample (10, 7.2%) and rigorous methodology (4, 2.9%) more frequently. Concerning the details of intervention implementation, GRADE CPG research priorities were more commonly related to dose (25, 3.7%), treatment time interval (19, 2.8%) and duration of therapy (16, 2.4%), while Chinese CPG research priorities were mostly related to dose (6, 4.3%), course of treatment (4, 2.9%) and treatment prescription (3, 2.2%) (Figs 2 and 3).

## Discussion

### Principal findings

This study reviewed 45 GRADE CPGs and 90 Chinese CPGs published in 2018 to 2022, and identified 668 and 138 research priorities, respectively. While 88.9% (40) of GRADE CPGs reported research priorities, only 28.9% (26) of Chinese CPGs did so. Analysis of the PICO elements of research priorities revealed that about 70% of both GRADE and Chinese research priorities had a PI structure. Furthermore, 14.8% (99) of GRADE CPG research priorities had PIC structures, compared to only 2.9% (4) in Chinese. Of notice, 28.4% (190) of GRADE CPG research priorities reflected comparisons between PICO elements, approximately double the proportion observed in Chinese research priorities. More than half of both GRADE and Chinese CPG research priorities focused on the dimensions of desirable or undesirable anticipated effects of the GRADE EtD framework. However, for the other dimensions, the concerns were different: the former was more focused on health equity and acceptability, while the latter was more concerned on feasibility and quality of evidence.

### Strength and limitations

In our study, we conducted a detailed analysis of the presentation form, structure and type of research priorities in GRADE CPGs and Chinese CPGs. To our knowledge, no comprehensive and systematic research of such research priorities before.

There were limitations in our study. We accepted and analyzed the research priorities reported in the CPGs without further investigating the appropriateness of the underlying logic of the prioritization. Additionally, in most cases, the presented research recommendations were due to the lack of evidence or the low-quality of evidence. Different guideline development groups may have differences or confusion about the definition of the lack of evidence and quality of evidence.

### Relation to previous work

A previous study formulated research priorities by developing a process, benefiting from considering the evidence base while identifying current knowledge gaps as well as any uncertainty [25]. Other studies focused on the structure of the research priorities, which summarized EPICOT (evidence, population, intervention, comparison, outcome, and time) as an essential structural element and emphasized the burden of disease and type of study as details that should be further considered [26–28]. Robinson and his colleagues identified 62 research gaps in five guidelines published by the Cystic Fibrosis Foundation and found that only 20% of the evidence gap were identified as research priorities by the guideline development group. They suggested that guideline developers should articulate research priorities more explicitly and systematically [24]. Although these studies explored research gaps or research priorities, they often conflated the two, which may not accurately reflect the connotation of the research priorities.

### Implications for research and guidelines

We emphasize the distinction between evidence gaps and research priorities as an independent dimension. Not all evidence gaps indicate a reason to be prioritized. Significant effort in enhancing the methods and standards of identifying and reporting of research priorities should be taken.

First of all, the key structure elements of research priorities should be reported as comprehensive and standardized as possible. Clinical question formulation frameworks such as the PICO or EPICOT models can facilitate the transformation of research priorities into clinical questions efficiently and accurately, ensuring consistency with the actual needs of clinical practice and future research. Secondly, attention should be directed towards addressing research priorities within the dimensions of the GRADE EtD framework, including health equity, feasibility, values and preferences. Thirdly, guideline development groups should be encouraged to standardize the description of research priorities in guideline reporting, positioning them consistently and independently whenever feasible to aid identification by guideline users. Lastly, more attention should be paid to identify research priorities on details of intervention implementation, which are essential for developing really practical clinical recommendations.

Besides the above, another issue needs to be clarified. The rationale for proposing research priorities should not be solely tied to evidence absence or low-quality. It would be of great value of we could point out research saturation areas to avoid research waste when there is high-quality evidence that is robust enough that very unlikely to be overturned in the future.

## Conclusion

There was still considerable room for improvement in the developing and reporting of research priorities in Chinese CPGs. Key PICO elements were inadequately presented, with more attention on intervention efficacy and insufficient consideration given to values, preferences, health equity, and feasibility. Identifying and reporting of research priorities deserves greater effort in the future.

## Supporting information

S1 File Search strategy. (DOCX)

S2 File Guidelines for inclusion. (DOCX)

S1 Fig Reported descriptions of research priorities in GRADE CPGs. (TIF)

S2 Fig Reported descriptions of research priorities in Chinese CPGs. (TIF)
