# Supplementary material for: Emphasis should be placed on identifying and reporting research priorities to increase research value: An empirical analysis
Source: PLoS One. 2024 Mar 22;19(3):e0300841. doi: 10.1371/journal.pone.0300841 (PMC10959327; doi:10.1371/journal.pone.0300841)
Supplement: S1 File — (DOCX) [file pone.0300841.s001.docx]

**Search Strategy**

### Database search of the Traditional Chinese Medicine clinical practice guidelines

| **Database** | **Search Strategy** | **References** |
| --- | --- | --- |
| PubMed | ("Practice Guideline"[Publication Type] OR "Guideline"[Publication Type] OR "Guidelines as Topic"[MeSH Terms] OR "clinical guidance"[Title/Abstract] OR "clinical practice guidance"[Title/Abstract] OR "guideline*"[Title/Abstract] OR "guidance*"[Title/Abstract] OR "clinical guidance"[Title/Abstract] OR "clinical guideline"[Title/Abstract] OR "evidence-based guideline"[Title/Abstract]) AND ((guideline[Filter] OR practice guideline[Filter]) AND (humans[Filter]) AND (2018/1/1:2022/12/31[pdat])) | 6267 |
| Embase | ('clinical practice guidance':ti,ab,kw OR 'guideline*':ti,ab,kw OR 'guidance*':ti,ab,kw OR 'clinical guidance':ti,ab,kw OR 'clinical guideline':ti,ab,kw OR 'evidence-based guideline':ti,ab,kw OR 'practice guideline'/exp) AND (2018:py OR 2019:py OR 2020:py OR 2021:py OR 2022:py) | 195084 |

We also searched four Chinese databases (China National Knowledge Infrastructure, VIP Database for Chinese Technical Periodicals, Wanfang, and Chinese Biomedical Literature Database), the Chinese database search strategy can be obtained by contacting us.

### Database search of the GRADE clinical practice guidelines

| **Database** | **Step** | **Search Strategy** | **References** |
| --- | --- | --- | --- |
| PubMed | #1 | (((((((((("GRADE Centre"[Title/Abstract]) OR("GRADE Centre"[Author])) OR ("GRADE Center"[Author])) OR ("GRADE working group"[Title/Abstract])) OR ("GRADE working group"[Author])) OR ("McMaster University GRADE Centre"[Title/Abstract])) OR ("McMaster University GRADE Centre"[Author])) OR (Gordon H. Guyatt[Author])) OR (Holger J. Schünemann[Author])) OR ("McMaster University GRADE Center"[Author])) OR ("McMaster University GRADE Center"[Title/Abstract]) | 1395 |
|  | #2 | (("Practice Guideline" [Publication Type] OR "Guideline" [Publication Type] OR "Guidelines as Topic"[Mesh]) OR ((((((("clinical guidance"[Title/Abstract]) OR ("clinical practice guidance"[Title/Abstract])) OR ("guideline*"[Title/Abstract])) OR ("guidance*"[Title/Abstract])) OR ("clinical guidance"[Title/Abstract])) OR ("clinical guideline"[Title/Abstract])) OR ("evidence-based guideline"[Title/Abstract]))) | 702974 |
|  | #3 | (("GRADE Centre"[Title/Abstract] OR "GRADE Centre"[Author] OR "GRADE Center"[Author] OR "GRADE working group"[Title/Abstract] OR "GRADE working group"[Author] OR "McMaster University GRADE Centre"[Title/Abstract] OR "McMaster University GRADE Centre"[Author] OR guyatt, gordon h[Author] OR (schunemann, holger j[Author] OR j schunemann, holger[Author]) OR "McMaster University GRADE Center"[Author]) AND ("Practice Guideline"[Publication Type] OR "Guideline"[Publication Type] OR "Guidelines as Topic"[MeSH Terms] OR ("clinical guidance"[Title/Abstract] OR "clinical practice guidance"[Title/Abstract] OR "guideline*"[Title/Abstract] OR "guidance*"[Title/Abstract] OR "clinical guidance"[Title/Abstract] OR "clinical guideline"[Title/Abstract] OR "evidence-based guideline"[Title/Abstract])) AND 2018/01/01:2022/12/31[Date - Publication]) AND (2018/1/1:2022/12/31[pdat]) | 275 |
| Embase | #1 | 'grade centre':ti,ab,kw OR 'grade centre':au OR 'grade center':au OR 'grade working group':ti,ab,kw OR 'grade working group':au OR 'mcmaster university grade centre':ti,ab,kw OR 'mcmaster university grade centre':au OR 'gordon h. guyatt':au OR 'holger j. schünemann':au OR 'mcmaster university grade center':ti,ab,kw OR 'mcmaster university grade center':au | 408 |
|  | #2 | 'clinical practice guidance':ti,ab,kw OR 'guideline*':ti,ab,kw OR 'guidance*':ti,ab,kw OR 'clinical guidance':ti,ab,kw OR 'clinical guideline':ti,ab,kw OR 'evidence-based guideline':ti,ab,kw OR 'practice guideline'/exp | 1266459 |
|  | #3 | ('grade centre':ti,ab,kw OR 'grade centre':au OR 'grade center':au OR 'grade working group':ti,ab,kw OR 'grade working group':au OR 'mcmaster university grade centre':ti,ab,kw OR 'mcmaster university grade centre':au OR 'gordon h. guyatt':au OR 'holger j. schünemann':au OR 'mcmaster university grade center':ti,ab,kw OR 'mcmaster university grade center':au) AND ('clinical practice guidance':ti,ab,kw OR 'guideline*':ti,ab,kw OR 'guidance*':ti,ab,kw OR 'clinical guidance':ti,ab,kw OR 'clinical guideline':ti,ab,kw OR 'evidence-based guideline':ti,ab,kw OR 'practice guideline'/exp) AND (2018:py OR 2019:py OR 2020:py OR 2021:py OR 2022:py) | 117 |

We also searched four Chinese databases (China National Knowledge Infrastructure, VIP Database for Chinese Technical Periodicals, Wanfang, and Chinese Biomedical Literature Database), the Chinese database search strategy can be obtained by contacting us.

### Websites search of the Chinese clinical practice guidelines

| **Institution** | **Webpage** |
| --- | --- |
| China Association of Chinese Medicine | https://www.cacm.org.cn/ |
| China Association of Traditional Chinese Medicine | http://www.caChinese.org.cn/ |
| Chinese Association of Integrative Medicine | http://www.caim.org.cn/ |
| China Association for Acupuncture and Moxibustion | http://www.caam.cn/ |
| World federation of Chinese medicine societies | http://www.wfcms.org/ |
| Doctor Society of integrative Medicine | http://www.cmda.net/ |
| Chinese Medical Association | https://www.cma.org.cn/?c=0 |
| Yimaitong | https://www.medlive.cn/ |
| Full-text Database of Chinese Medical Journals | https://www.yiigle.com/index |
