# Supplementary material for: Emphasis should be placed on identifying and reporting research priorities to increase research value: An empirical analysis
Source: PLoS One. 2024 Mar 22;19(3):e0300841. doi: 10.1371/journal.pone.0300841 (PMC10959327; doi:10.1371/journal.pone.0300841)
Supplement: S2 File — (DOCX) [file pone.0300841.s002.docx]

**Guidelines for inclusion**

| **Number** | **Title of clinical practice guideline** | **Country/region** | **Developer** | **Year** |
| --- | --- | --- | --- | --- |
|  |  |  |  |  |
| 1 | Chinese Clinical Practice Guidelines in Treating  Knee Osteoarthritis by Periarticular Knee Osteotomy | China | Chinese Medical Doctor Association, Chinese Medical Association of Orthopaedic Doctor Association, Chinese Orthopaedic Doctor Association | 2022 |
| 2 | Acupuncture for cancer pain:  an evidence-based clinical practice guideline | China | / | 2022 |
| 3 | Clinical Guidelines for Diagnosis and Treatment of Frozen Shoulder in Traditional Chinese Medicine | China | China Society of Traditional Chinese Medicine | 2022 |
| 4 | American Society of Hematology living guidelines on the use of  anticoagulation for thromboprophylaxis for patients with COVID-19:  March 2022 update on the use of anticoagulation in critically ill patients | America | The American Society of Hematology | 2022 |
| 5 | Plasma exchange and glucocorticoid dosing for patients with  ANCA-associated vasculitis: a clinical practice guideline | International group | BMJ | 2022 |
| 6 | 2021 American College of Rheumatology/Vasculitis Foundation  Guideline for the Management of Kawasaki Disease | America | The American College of Rheumatology / Vasculitis Foundation | 2022 |
| 7 | Endovascular thrombectomy and intravenous alteplase in  patients with acute ischemic stroke due to large vessel  occlusion: A clinical practice guideline | China | / | 2022 |
| 8 | Guidelines for the prevention and management of children  and adolescents with COVID‑19 | China | / | 2022 |
| 9 | Joint 2022 European Society of Thoracic Surgeons and The American  Association for Thoracic Surgery guidelines for the prevention of  cancer-associated venous thromboembolism in thoracic surgery | Europe | The European Society of Thoracic Surgeons | 2022 |
| 10 | American Society of Hematology, ABHH, ACHO, Grupo CAHT,  Grupo CLAHT, SAH, SBHH, SHU, SOCHIHEM, SOMETH, Sociedad Paname~na de Hematologıa, Sociedad Peruana de Hematologıa, and SVH 2022 guidelines for prevention of venous thromboembolism in surgical and medical patients and long-distance travelers in Latin America | America | The American Society of Hematology | 2022 |
| 11 | World Allergy Organization (WAO) Diagnosisand Rationale for Action against Cow’s MilkAllergy (DRACMA) Guideline update – XIV –Recommendations on CMA immunotherapy | International group | The World Allergy Organization (WAO) | 2022 |
| 12 | 2022 Chinese national clinical practice guideline on Helicobacter  pylori eradication treatment | China | multidisciplinary group | 2022 |
| 13 | Integrated guidelines for the clinical management of premalignant gastric cancer lesions in China | China | Spleen and stomach Disease Branch of Chinese Association of Traditional Chinese Medicine | 2022 |
| 14 | Guidelines for perioperative pain management in total knee arthroplasty in China (2022) | China | Joint Surgery Group of Bone Science Branch of Chinese Medical Association, Joint Surgery Group of Orthopaedic Professional Committee of Beijing Medical Association | 2022 |
| 15 | Clinical practice guidelines for hypertension in China | China | The Chinese Medical Doctor Association of the National Center for Cardiovascular Disease | 2022 |
| 16 | Clinical management guidelines for acute fatty liver of pregnancy in China ( 2022) | China | Obstetrics and Science Group of Obstetrics and Gynecology Branch of Chinese Medical Association | 2022 |
| 17 | Evidence-based guidelines for active screening of tuberculosis in China | China | Tuberculosis Control Professional Branch of China Tuberculosis Association | 2022 |
| 18 | Clinical practice guidelines for the rehabilitation of SCI | China | Professional Committee of Spinal Cord Injury Rehabilitation of Chinese Rehabilitation Association for Disabled Persons | 2022 |
| 19 | Guideline for clinical evidence-based diagnosis and treatment of Achilles tendon rupture | China | Trauma and Bone Science Group, Bone Science Branch of Chinese Medical Association | 2022 |
| 20 | Chinese guideline for the secondary prevention of ischemic stroke and transient ischemic  attack 2022 | China | Neurology Society of Chinese Medical Association | 2022 |
| 21 | International TCM Clinical Practice Guide for Glycoipid Metabolism | China | The World Federation of Traditional Chinese Medicine Societies | 2022 |
| 22 | Chinese patent medicine treats idiopathic less, weak sperm male infertility Guidelines for clinical application | China | China Association of Traditional Chinese Medicine | 2022 |
| 23 | Guidelines for Clinical Application of proprietary Chinese Medicine for Breast Hyperplasia (2021 Edition) | China | China Association of Traditional Chinese Medicine | 2022 |
| 24 | Guidelines for the clinical application of proprietary Chinese medicine for treating chronic prostatitis | China | China Association of Traditional Chinese Medicine | 2022 |
| 25 | Guidelines for the clinical application of proprietary Chinese patent medicine in the treatment of osteoporosis | China | China Association of Traditional Chinese Medicine | 2022 |
| 26 | TCM intervention after conventional treatment of colorectal cancer fingerpost | China | China Society of Traditional Chinese Medicine, Chinese Society of Ethnic Medicine | 2022 |
| 27 | Intervention program for asthma treatment | China | China Association of Traditional Chinese Medicine | 2022 |
| 28 | Intervention program for chronic obstructive pulmonary disease treatment | China | China Association of Traditional Chinese Medicine | 2022 |
| 29 | Guide for TCM diagnosis and treatment of periinflammation of shoulder | China | China Association of Traditional Chinese Medicine | 2022 |
| 30 | Chinese Medicine Guideline for Diagnosis and Treatment of Osteoporosis Fracture | China | China Association of Traditional Chinese Medicine | 2022 |
| 31 | Evidence-based practice guidelines for the clinical rehabilitation of chronic obstructive pulmonary disease | China | Evidence-based Rehabilitation Medical Affairs Working Committee of Chinese Rehabilitation Medical Association | 2021 |
| 32 | Cough Guidelines for Chinese Children (2021 Patient Edition) | China | Clinical Pharmacology Group, Pediatrics Branch of Chinese Medical Association, National Clinical Medical Research Center for Child Health and Disease | 2021 |
| 33 | Chinese guideline for diagnosis and treatment of osteoarthritis (2021 edition) | China | Joint Surgery Group, Bone Science Branch of Chinese Medical Association | 2021 |
| 34 | Evidence-based guidelines for the clinical practice of intravenous fluid therapy in children | China | Evidence-based guidelines for the clinical practice of intravenous fluid therapy in children | 2021 |
| 35 | Chinese guidelines for the management of women with epilepsy during periconceptional period | China | Electroencephalography and Epileptsy Group of Neurology Branch of Chinese Medical Association | 2021 |
| 36 | Evidence-based medical guidelines for the treatment of early motor symptoms in Parkinson's disease in China | China | Parkinson's Disease and Dyskinesia Group of Neurology Branch of Chinese Medical Association | 2021 |
| 37 | Evidence-based guidelines for the treatment of Retinitis pigmentosa (2021) | China | China Eye Genetic Disease Diagnosis and Treatment Group of China Eye Genetic Alliance, Genetic Ophthalmology Group of Ophthalmologist Branch of Chinese Medical Doctor Association | 2021 |
| 38 | Clinical practice guidelines for diagnosis and treatment of lumbar disc herniation in the elderly | China | Orthopaedic Rehabilitation Group, Orthopedics Branch of Chinese Medical Association Lumbar Research Group, Spinal Spinal cord Professional Committee of Chinese Rehabilitation Medical Association | 2021 |
| 39 | Chinese guidelines for diagnosis of Creutzfeldt‑Jakob disease 2021 | China | Neurological Infectious Diseases and Cerebrospinal fluid Cytology Group of Neurology Branch of Chinese Medical Association | 2021 |
| 40 | Diabetic retinopathy syndrome combined with the diagnosis and treatment guidelines | China | Integrated Traditional Chinese and Western Medicine Doctor Branch of Chinese Medical Doctor Association | 2021 |
| 41 | Hyperthyroidism syndrome combined with the diagnosis and treatment guidelines | China | ntegrated Traditional Chinese and Western Medicine Doctor Branch of Chinese Medical Doctor Association | 2021 |
| 42 | Type 2 diabetes disease syndrome combined with the diagnosis and treatment guidelines | China | Integrated Traditional Chinese and Western Medicine Doctor Branch of Chinese Medical Doctor Association | 2021 |
| 43 | Guidelines for the clinical application of proprietary Chinese medicine in the treatment of vascular dementia | China | China Association of Traditional Chinese Medicine | 2021 |
| 44 | Guidelines for the clinical application of proprietary Chinese patent medicine for eczema treatment | China | China Association of Traditional Chinese Medicine | 2021 |
| 45 | Clinical practice guidelines for TCM rehabilitation · Incomplete paraplegia | China | Other / by the TCM rehabilitation standard research base | 2021 |
| 46 | Evidence-based clinical guidelines for massage therapy for the treatment of acute diarrhea in children | China | / | 2021 |
| 47 | Guidelines for the clinical diagnosis and treatment of integrated Traditional Chinese and Western medicine --Tic disorder | China | China Association of Traditional Chinese Medicine | 2021 |
| 48 | Traditional Chinese medicine clinical diagnosis and treatment guidelines of plateau polycythemia | China | China Association of Traditional Chinese Medicine | 2021 |
| 49 | Diagnosis and treatment guideline for Chinese medicine on  acute trachea-bronchitis | China | China Association of Traditional Chinese Medicine | 2021 |
| 50 | Clinical practice guidelines for the treatment of allergic  rhinitis in children with traditional Chinese medicine | China | / | 2021 |
| 51 | Medical cannabis or cannabinoids for chronic pain: a clinical practice  guideline | International group | BMJ | 2021 |
| 52 | A living WHO guideline on drugs to prevent covid-19 | WHO | BMJ | 2021 |
| 53 | SGLT-2 inhibitors or GLP-1 receptor agonists for adults with type 2  diabetes: a clinical practice guideline | International group | BMJ | 2021 |
| 54 | 2021 American College of Rheumatology/Vasculitis  Foundation Guideline for the Management of  Polyarteritis Nodosa | America | The American College of Rheumatology / Vasculitis Foundation | 2021 |
| 55 | 2021 American College of Rheumatology/Vasculitis  Foundation Guideline for the Management of  Antineutrophil Cytoplasmic Antibody–Associated Vasculitis | America | The American College of Rheumatology / Vasculitis Foundation | 2021 |
| 56 | 2021 American College of Rheumatology/Vasculitis  Foundation Guideline for the Management of Giant Cell  Arteritis and Takayasu Arteritis | America | The American College of Rheumatology / Vasculitis Foundation | 2021 |
| 57 | 2019 American College of Rheumatology/Arthritis  Foundation Guideline for the Management of  Osteoarthritis of the Hand, Hip, and Knee | America | The American College of Rheumatology / Vasculitis FoundationAssociation. | 2021 |
| 58 | 2021 Guidelines for VTE management in Latin America | America | American Society of Hematology, 12 Hematology Societies in Latin America | 2021 |
| 59 | ASH ISTH NHF WFH 2021 guidelines on the management of von  Willebrand disease | America | The American Society of Hematology (ASH), the International Society for Thrombosis and Hemostasis (ISTH), the National Hemophilia Foundation (NHF), and the World Hemophilia Federation (WFH) | 2021 |
| 60 | Canadian Rheumatology Association Recommendation for the  Use of COVID-19 Vaccination for Patients With Autoimmune  Rheumatic Diseases | Canada | The Canadian Society of Rheumatology | 2021 |
| 61 | Clinical practice guidelines for the exercise therapy of knee osteoarthritis | China | Writing group of clinical practice guidelines for exercise treatment of knee osteoarthritis | 2020 |
| 62 | Clinical pain management of osteoarthritis in China Practice Guide (2020 Edition) | China | Joint Surgery Group, Bone Science Branch of Chinese Medical Association | 2020 |
| 63 | Chinese guidelines for perioperative airway management in thoracic surgery (2020  edition) | China | Compilation Committee of Chinese Guidelines for Perioperative Airway Management in Thoracic Surgery (2020 edition)of integrative Medicine, Chinese Medical Doctor Association. | 2020 |
| 64 | Intestinal preparation related to digestive endoscopy diagnosis and treatment in Chinese children is fast fingerpost | China | Pediatric Collaborative Group of Digestive Endoscopy Society of Chinese Medical Association | 2020 |
| 65 | Chinese Guidelines for Diagnosis and Treatment of Patellofemoral Osteoarthritis (2020) | China | National Clinical Medical Research Center for Geriatric Diseases (Xiangya Hospital) | 2020 |
| 66 | Clinical guidelines for the diagnosis and treatment of feeding intolerance in preterm infants (2020) | China | Evidence-based Professional Committee of Neonatologist Branch of Chinese Medical Doctor Association | 2020 |
| 67 | Guidelines for the clinical application of lumbar oblique lateral interbody fusion | China | Spinal Surgery Group, Orthopedics Branch of Chinese Medical Association | 2020 |
| 68 | Guidelines for the Diagnosis and Treatment of Lower Gastrointestinal Bleeding (2020) | China | Colorectal group of Digestive Endoscopy Branch of Chinese Medical Society | 2020 |
| 69 | Guidelines for Clinical Management of Twin Pregnancy (2020) | China | Fetal Medicine Group, Perinatal Medicine Branch of Chinese Medical Association | 2020 |
| 70 | Guidelines for multidisciplinary diagnosis and treatment of hypertensive cerebral hemorrhage in China | China | The Neurosurgery Branch of the Chinese Medical Association | 2020 |
| 71 | Chinese guidelines for diagnosis and treatment of essential tremor 2020 | China | Parkinson's Disease and Dyskinesia Group of Neurology Branch of Chinese Medical Association | 2020 |
| 72 | Guidelines for the diagnosis and treatment of integrated traditional Chinese and Western medicine for tubal pregnancy | China | Chinese Association of the Integration of Traditional and Western Medicine | 2020 |
| 73 | Guidelines for the diagnosis and treatment of integrated traditional Chinese and Western medicine for abnormal uterine bleeding | China | Chinese Association of the Integration of Traditional and Western Medicine | 2020 |
| 74 | International TCM Clinical Practice Guidelines for rheumatoid arthritis | China | The World Federation of Traditional Chinese Medicine Societies and the China Association of Traditional Chinese Medicine | 2020 |
| 75 | TCM Clinical Practice Guidelines for migraines | China | / | 2020 |
| 76 | Guidelines for clinical diagnosis and treatment of Traditional Chinese medicine · Cransolution (Pediatric hydrocephalus) | China | China Association of Traditional Chinese Medicine | 2020 |
| 77 | Gastrointestinal bleeding prophylaxis  for critically ill patients: a clinical practice  guideline | International group | BMJ | 2020 |
| 78 | 2020 American College of Rheumatology Guideline for the  Management of Gout | America | The American College of Rheumatology | 2020 |
| 79 | 2020 American College of Rheumatology Guideline for the  Management of Reproductive Health in Rheumatic and  Musculoskeletal Diseases | America | The American College of Rheumatology | 2020 |
| 80 | European guidelines on breast cancer screening and diagnosis | Europe | European Commission | 2020 |
| 81 | American Society of Hematology 2020 guidelines for treating newly  diagnosed acute myeloid leukemia in older adults | America | The American Society of Hematology | 2020 |
| 82 | American Society of Hematology 2020 guidelines for management of venous thromboembolism: treatment of deep vein thrombosis and pulmonary embolism | America | The American Society of Hematology | 2020 |
| 83 | Transfusion strategies in non-bleeding  critically ill adults: a clinical practice guideline  from the European Society of Intensive Care  Medicine | Europe | The European Society of Intensive Care Medicine | 2020 |
| 84 | Remdesivir for severe covid-19: a clinical practice guideline | International group | BMJ | 2020 |
| 85 | Guidelines for the diagnosis and treatment of CNS tuberculosis in China | China | Professional Committee of Tuberculosis Society of Chinese Medical Association | 2019 |
| 86 | Clinical guidelines for the diagnosis and treatment of OI | China | The Osteoporosis and Bone Mineral Salt Disease Branch of the Chinese Medical Association | 2019 |
| 87 | Chinese guidelines for the diagnosis and treatment of androgen alopecia | China | Hair Plastic Surgery Professional Committee of Beauty and Plastic surgeon Branch of Chinese Medical Doctor Association | 2019 |
| 88 | Guidelines for diagnosis and treatment of anemia during perioperative period of  orthopedic surgery in China | China | Professional Committee of Musculoskeletal Sports Rehabilitation Technology Transformation of China Rehabilitation Technology Transformation and Development Promotion Association | 2019 |
| 89 | Clinical practice guidelines for the diagnosis and treatment of laryngomalacia in children | China | Pediatric Otolaryngology Group, Minimally Invasive Branch of Chinese Maternal and Child Health Society | 2019 |
| 90 | Chinese Evidence-based Clinical Practice Guidelines for Stroke (2019 Edition) | China | Rehabilitation Treatment Group of Physical Medicine and Rehabilitation Branch of Chinese Medical Association | 2019 |
| 91 | Clinical practice guidelines for omalizumab in the treatment of childhood allergic asthma | China | Multidisciplinary group | 2019 |
| 92 | Guidelines for the prevention and treatment of hepatitis C (2019 version) | China | The Liver Disease Society of the Chinese Medical Association | 2019 |
| 93 | Guidelines for surgical treatments of metastatic spinal tumors | China | Bone Oncology Group, Bone Science Branch of the Chinese Medical Association | 2019 |
| 94 | Clinical guidelines on nutrition in end － stage liver disease | China | Liver Society of Chinese Medical Association, Gastroenterology Society of Chinese Medical Association | 2019 |
| 95 | Guidelines for the diagnosis and treatment of integrated traditional Chinese and Western medicine in endometriosis | China | Chinese Association of the Integration of Traditional and Western Medicine | 2019 |
| 96 | Guidelines for the diagnosis and treatment of integrated Traditional Chinese and Western medicine for osteoporosis | China | Chinese Association of the Integration of Traditional and Western Medicine | 2019 |
| 97 | ruffian TCM Clinical Practice Guide (2018) | China | The World Federation of Traditional Chinese Medicine Societies | 2019 |
| 98 | The International TCM Clinical Practice Guide for forgetfulness | China | The World Federation of Traditional Chinese Medicine Societies | 2019 |
| 99 | Osteoarthritis syndrome combined with the diagnosis and treatment guidelines | China | China Association of Traditional Chinese Medicine | 2019 |
| 100 | Clinical diagnosis and treatment guidelines of Traditional Chinese Medicine: Knei disease (knee osteoarthritis) | China | China Association of Traditional Chinese Medicine | 2019 |
| 101 | Evidence-based guidelnes of cinical practice wth acupunctre and moxibustion Pain after anke sprain | China | China Association of Acupuncture and Moxibustion | 2019 |
| 102 | Evidence-based guidelines of clinical practice with acupuncture and moxibustion Tenosynovitis pain | China | China Association of Acupuncture and Moxibustion | 2019 |
| 103 | Evidence-based guidelines of clinical practice with acupuncture and moxibustion Hypochondriac pain | China | China Association of Acupuncture and Moxibustion | 2019 |
| 104 | Evidence-based guidelines of clical practice with Acupuncture and moxibustion istending pain caused by lower extremity varicose veins | China | China Association of Acupuncture and Moxibustion | 2019 |
| 105 | Colorectal cancer screening with faecal immunochemical testing sigmoidoscopy or colonoscopy: a clinical practice guideline | International group | BMJ | 2019 |
| 106 | American Society of Hematology 2019 guidelines for management of  venous thromboembolism: prevention of venous thromboembolism in  surgical hospitalized patients | America | American Society of Blood | 2019 |
| 107 | Treatment of patients with nonsevere and  severe coronavirus disease 2019: an evidencebased guideline | China | International group | 2019 |
| 108 | Subacromial decompression surgery  for adults with shoulder pain: a clinical  practice guideline | International group | BMJ | 2019 |
| 109 | Guidelines for the diagnosis and treatment of cirrhotic ascites and related complications | China | The Liver Disease Society of the Chinese Medical Association | 2018 |
| 110 | Clinical evidence-based diagnosis and treatment guidelines of giant cell tumor of bone | China | Bone Tumor Professional Committee of Orthopedics Branch of Chinese Medical Doctor Association | 2018 |
| 111 | Joint cavity injection of platelet-rich plasma to treat knee osteoarthritis Clinical Practice Guidelines (2018 edition) | China | Orthopaedic Branch of China Medical Care International Exchange Promotion Association | 2018 |
| 112 | Clinical practice guidelines for liver transplantation for liver cancer in China (Version 2018 edition) | China | The Organ Transplant Physician Branch of the Chinese Medical Doctor Association | 2018 |
| 113 | Recommended guidelines for the clinical treatment of keloid lesions in China | China | Expert group of standing Committee of scar Medicine Branch of China Plastic Surgery Association | 2018 |
| 114 | Treatment and surveillance for non-muscle-invasive bladder cancer in China: an  evidence-based clinical practice guideline | China | Professional Committee of Urology of the Chinese Society of Research Hospitals | 2018 |
| 115 | 2018 China Guideline for the Diagnosis and Treatment of Senile Osteoporosis | China | The Working Group of The Guidelines for the Diagnosis and Treatment of Osteoporosis in the Elderly in China (2018) | 2018 |
| 116 | Guidelines for Diagnosis and Treatment of Integrated Chinese and Western Medicine (Trial Edition) | China | Chinese Association of the Integration of Traditional and Western Medicine | 2018 |
| 117 | Guidelines for the integration of traditional Chinese and Western medicine for the diagnosis and treatment of acute myocardial infarction | China | Integrated Traditional Chinese and Western Medicine Doctor Branch of Chinese Medical Doctor Association | 2018 |
| 118 | Guidelines for TCM diagnosis and treatment of community-acquired pneumonia | China | China Society of Traditional Chinese Medicine, Chinese Society of Ethnic Medicine | 2018 |
| 119 | Guidelines for TCM diagnosis and treatment before and after coronary heart disease and angina pectoris intervention | China | China Association of Traditional Chinese Medicine | 2018 |
| 120 | Rheumatoid arthritis syndrome combined with the diagnosis and treatment guidelines | China | China Association of Traditional Chinese Medicine | 2018 |
| 121 | Atraumatic (pencil-point) versus conventional needles for lumbar  puncture: a clinical practice guideline | International group | BMJ | 2018 |
| 122 | Antibiotics after incision and drainage for  uncomplicated skin abscesses: a clinical  practice guideline | International group | BMJ | 2018 |
| 123 | Patent foramen ovale closure, antiplatelet  therapy or anticoagulation therapy alone  for management of cryptogenic stroke?  A clinical practice guideline | International group | BMJ | 2018 |
| 124 | Corticosteroid therapy for sepsis: a clinical  practice guideline | International group | BMJ | 2018 |
| 125 | Prostate cancer screening with prostate-specific antigen (PSA) test:  a clinical practice guideline | International group | BMJ | 2018 |
| 126 | Evidence-based guidelines for supportive care of patients with  Ebola virus disease | / | / | 2018 |
| 127 | 2018 American College of Rheumatology/National Psoriasis  Foundation Guideline for the Treatment of Psoriatic Arthritis | America | The American College of Rheumatology | 2018 |
| 128 | American Society of Hematology 2018 guidelines for management of  venous thromboembolism: diagnosis of venous thromboembolism | America | The American Society of Hematology | 2018 |
| 129 | American Society of Hematology 2018 guidelines for management of  venous thromboembolism: prophylaxis for hospitalized and  nonhospitalized medical patients | America | The American Society of Hematology | 2018 |
| 130 | American Society of Hematology 2018 Guidelines for management of  venous thromboembolism: treatment of pediatric  venous thromboembolism | America | The American Society of Hematology | 2018 |
| 131 | American Society of Hematology 2018 guidelines for management of  venous thromboembolism: venous thromboembolism in the context  of pregnancy | America | The American Society of Hematology | 2018 |
| 132 | American Society of Hematology 2018 guidelines for management of  venous thromboembolism: optimal management of  anticoagulation therapy | America | The American Society of Hematology | 2018 |
| 133 | American Society of Hematology 2018 guidelines for management of venous thromboembolism: heparin-induced thrombocytopenia | Amercia | The American Society of Hematology | 2018 |
| 134 | Oxygen therapy for acutely ill medical patients: a clinical practice guideline | International group | BMJ | 2018 |
| 135 | Dual antiplatelet therapy with aspirin and clopidogrel for acute high risk transient ischaemic attack and minor ischaemic stroke: a clinical practice guideline | International group | BMJ | 2018 |
