# Supplementary figures and images for: Emphasis should be placed on identifying and reporting research priorities to increase research value: An empirical analysis
Source: PLoS One. 2024 Mar 22;19(3):e0300841. doi: 10.1371/journal.pone.0300841 (PMC10959327; doi:10.1371/journal.pone.0300841)

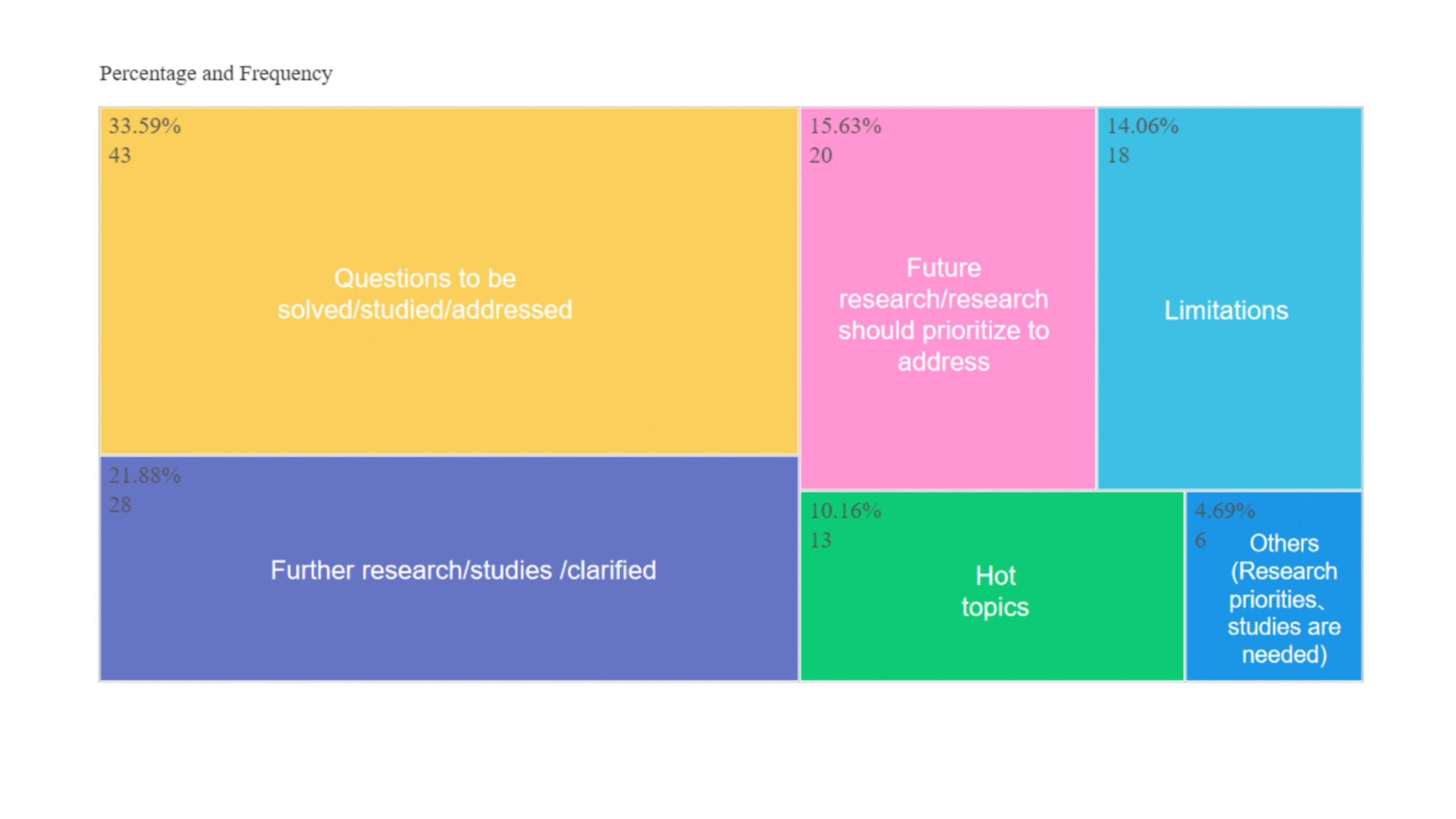

Supplement: S1 Fig — (TIF) [file pone.0300841.s003.tif]

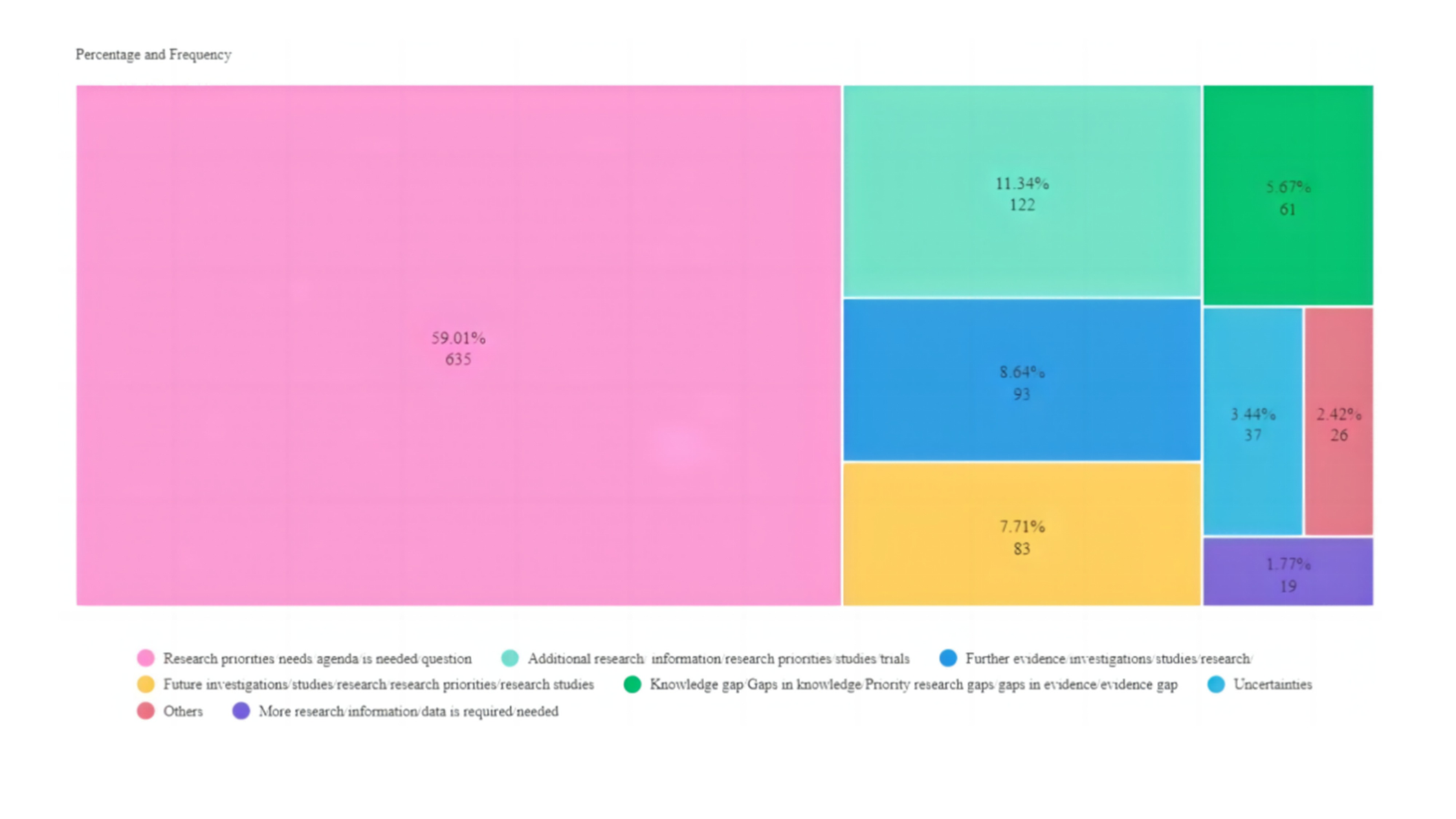

Supplement: S2 Fig — (TIF) [file pone.0300841.s004.tif]
